# Oxytetracycline-hydrocortisone ointment reduces the occurrence of both dry socket and post-extraction pain after third molar extraction: An observational study

**DOI:** 10.1371/journal.pone.0254221

**Published:** 2021-07-02

**Authors:** Hiroki Otake, Yoko Sato, Eiji Nakatani, Philip Hawke, Shingo Takei, Akihiko Ogino, Hideaki Asai, Atsushi Abe, Kohta Fukuta, Moriyasu Adachi

**Affiliations:** 1 Department of Oral and Maxillofacial Surgery, Shizuoka General Hospital, Shizuoka, Japan; 2 Research Support Center, Shizuoka General Hospital, Shizuoka, Japan; 3 School of Pharmaceutical Sciences, University of Shizuoka, Shizuoka, Japan; 4 Department of Oral and Maxillofacial Surgery, Holy Spirit Hospital, Nagoya, Japan; 5 Department of Oral and Maxillofacial Surgery, Nagoya Ekisaikai Hospital, Nagoya, Japan; Ohio State University Wexner Medical Center Department of Surgery, UNITED STATES

## Abstract

**Objectives:**

Dry socket and post-extraction pain are typical discomforts experienced by patients after tooth extraction. In this study, we inserted gauze coated with oxytetracycline-hydrocortisone ointment into the extraction socket immediately after lower third molar extraction and then evaluated the occurrence of dry socket and post-extraction pain compared with gauze non-insertion.

**Methods:**

This retrospective study was carried out on patients undergoing lower third molar extraction in the Department of Oral Surgery at Shizuoka Prefectural General Hospital in Shizuoka, Japan from November 2018 to October 2019. A comparison was carried out between a gauze-insertion group and a non-insertion group. The occurrence versus non-occurrence of dry socket was determined, and degree of pain was assessed based on a visual analogue scale (VAS) and on patients reporting the number of loxoprofen sodium oral analgesic tablets (60mg/tablet) that they had taken. Dry socket was defined as patient-reported spontaneous pain that did not subside 1 to 3 days postoperatively. Spontaneous post-extraction pain was recorded four times: on the operative day, on the first postoperative day (POD1), on POD3, and during suture removal (POD7).

**Results:**

The occurrence of dry socket was lower in the gauze-insertion group than in the non-insertion group (0.9%, 2/215 vs. 19.6%, 9/46, p<0.001). The results also showed that both VAS-defined pain level and the number of analgesic tablets taken were lower in the gauze-insertion group than in the non-insertion group on POD3 and POD7.

**Conclusions and clinical relevance:**

Inserting gauze coated with oxytetracycline-hydrocortisone ointment into the extraction socket immediately after third molar extraction reduces the occurrence of both dry socket and post-extraction pain.

## Introduction

Dry socket occurs when a clot becomes partially or completely detached from the extraction site before the wound has healed [1, 2]. It is one of the most studied complications in the field of oral and maxillofacial surgery [3]. Dry socket causes severe spontaneous pain 1 to 3 days after extraction, which may delay healing of the socket and sometimes progresses to chronic bone infection. The incidence of dry socket has been reported to be about 3% for all teeth and more than 30% for lower third molars [4]. Several studies have reported that a variety of local and systemic factors are involved in dry socket, but its exact etiology has not been clarified. Risk factors include male sex, smoking, and a history of inflammation [5, 6].

The methods of preventing dry socket that have been studied include administering chlorhexidine gel [7] or platelet-rich fibrin (PRF) [8] into the extraction socket, and rinsing the mouth with chlorhexidine mouthwash [7]. However, in our experience, these methods have drawbacks. Chlorhexidine gel easily flows out of the extraction socket and excessive rinsing of the mouth can lead to the loss of blood clots, reducing the effectiveness of these methods. PRF requires considerable time to prepare, making it an inefficient treatment method.

Some research has focused on the most common treatment for dry socket, which involves inserting ointment containing antibacterial or steroidal agents into the extraction socket and then covering the site with a surgical pack [9]. A study in which chlortetracycline ointment was applied to the socket with gauze immediately after tooth extraction showed a preventive effect on dry socket, but not on pain [10].

Hydrocortisone is an adjuvant used in complex surgical procedures that is well known for its potent anti-inflammatory properties [11]. In the present study, we investigated the effect of inserting gauze coated with oxytetracycline-hydrocortisone ointment into the socket immediately after third molar extraction on the occurrence of dry socket and spontaneous post-extraction pain.

## Method

### Study and participants

This retrospective study was carried out on patients who underwent lower third molar extraction in the Department of Oral Surgery at Shizuoka Prefectural General Hospital from November 2018 to October 2019.

Two groups of patients were studied. In the gauze-insertion group, surgical gauze containing X-ray contrast medium (Mekkin Komegaaze X, Osaki Medical, Aichi, Japan) was coated with oxytetracycline-hydrocortisone ointment (Terra-Cortril Ointment®, Yoshindo, Toyama, Japan) and inserted into the socket immediately after tooth extraction, followed by suturing. The non-insertion group did not receive this treatment prior to suturing. Gauze containing X-ray contrast medium was used in order to allow the gauze to be identified by imaging in the event that it came loose from the socket and was accidentally swallowed. From November 2018 to March 2019, the decision whether to insert gauze was made independently by the attending physician for clinical need; however, from April 2019, only gauze-insertion procedures were performed. Patients were excluded from analysis if they had simultaneous extraction of adjacent teeth or if they had taken medication for the extraction site prescribed by a physician outside our hospital.

This study was approved by the ethics committee of Shizuoka General Hospital (SGHIRB#2019013). The procedure in which gauze coated with oxytetracycline-hydrocortisone ointment is inserted into the socket immediately after extraction was certified by the Clinical Ethics Committee for Medical Procedures of Shizuoka General Hospital based on the 1980 notification on off-label use from the Director of the Health Insurance Bureau at the Ministry of Health, Labour and Welfare of Japan [12].

All data were fully anonymized before being accessed for this study. For retrospective studies using anonymized data, the Japanese Ethical Guidelines for Medical and Health Research Involving Human Subjects do not require information on the nature of future data disclosure to be included in informed consent agreements. Following these guidelines, the purpose and methods of this study were disclosed to potential participants on the hospital website, along with the recognition of their right to decline to participate in the study. Therefore, due to ethical limitations, the raw data for this study cannot be made freely available in the manuscript, supporting information files, or public repositories. Researchers seeking to access the dataset can file a request with the Clinical Trials Management Office at Shizuoka General Hospital (4-27-1 Kita-ando, Aoi-ku, Shizuoka, 420–8527, Japan. E-mail: chiken-sougou@shizuoka-pho.jp). All requests must be accompanied by a plan detailing the analysis to be carried out on the data and will be subject to approval by the hospital’s Steering Committee.

### Surgical procedure and insertion of gauze coated with oxytetracycline-hydrocortisone ointment

**Fig 1:**
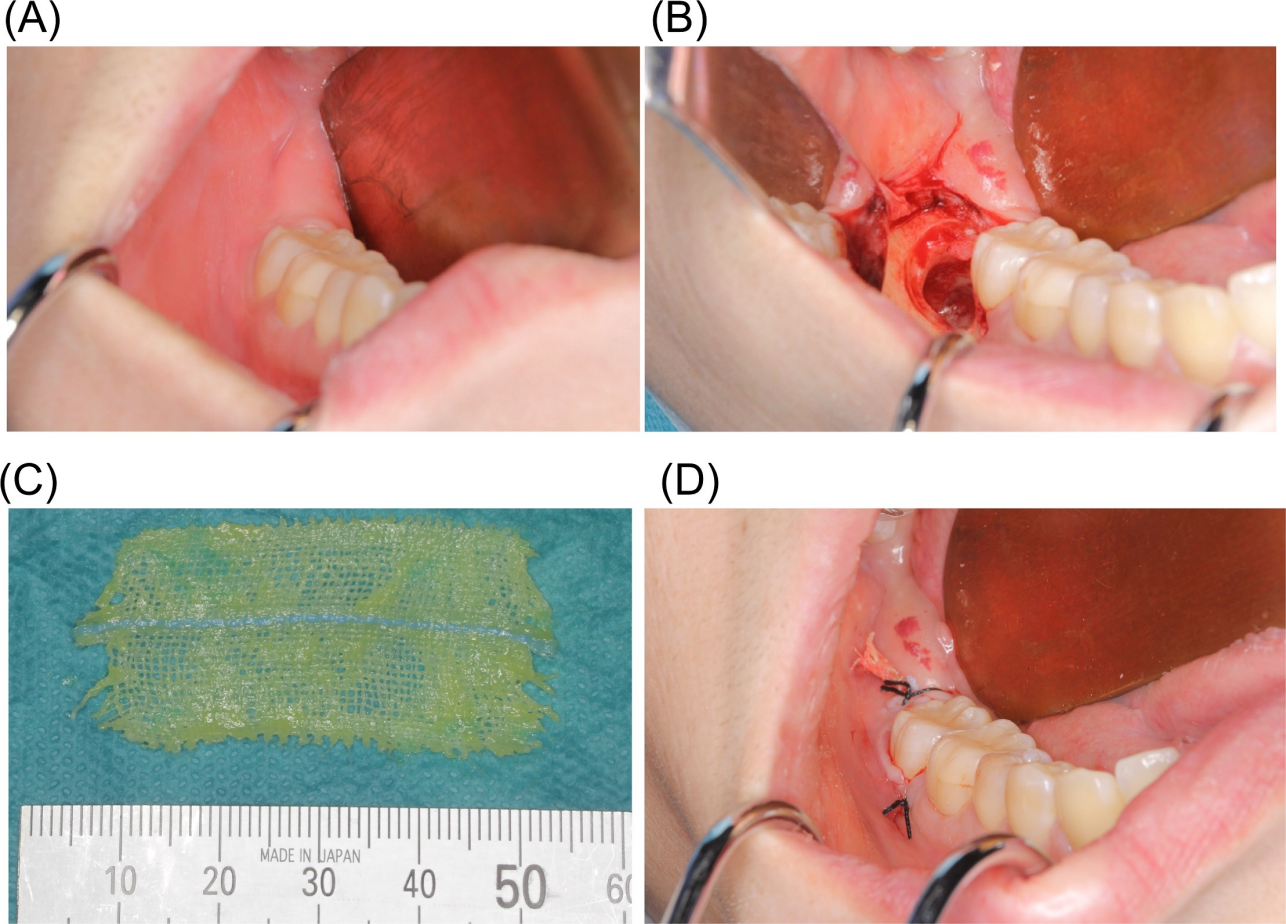
Surgical procedure and application of gauze coated with oxytetracycline-hydrocortisone ointment. (A) Preoperative view before lower third molar extraction under local anesthesia. (B) View after tooth extraction, curettage of the extraction socket, and cleansing with saline. (C) 5 x 2 cm piece of gauze coated with oxytetracycline-hydrocortisone ointment applied to the gauze-insertion group. (D) View after inserting gauze into socket and suturing.

All procedures were carried out by oral surgeons who had at least 5 years of experience and who had been certified by the Japanese Society of Oral and Maxillofacial Surgeons. Patients received two 500 mg tablets of amoxicillin orally 1 hour before surgery, then had a lower third molar extracted under local anesthesia. The inferior alveolar, long buccal, and lingual nerves were locally anesthetized with 2% lidocaine hydrochloride with epinephrine 1:200,000 (Xylocaine, Dentsply Sirona, Tokyo, Japan) (Fig 1A). A full-thickness Ward’s incision was placed, and the mucoperiosteal flap was reflected. After adequate bone removal, the tooth was removed, followed by curettage of the socket and cleansing with saline (Fig 1B). The flap was repositioned and closed with 3–0 silk suture in an interrupted fashion on the control side. Next, in the gauze-insertion group, 1 g of oxytetracycline-hydrocortisone ointment (containing 30 mg of oxytetracycline and 10 mg of hydrocortisone) was applied to a 5 x 2 cm piece of gauze (Fig 1C), and the gauze was inserted into the extraction socket to prevent the socket from falling out (Fig 1D). The amount of ointment used and the size of the gauze were standardized regardless of the size of the socket. Patients in both groups took amoxicillin 1500 mg/day orally for 2 days after the operation. For pain, one 60 mg tablet of loxoprofen sodium was taken orally at an interval of at least 6 hours when patients had pain. Sutures were removed on postoperative days 6 to 8. The gauze was removed at the same time as the sutures.

### Clinical parameters and questionnaire

G.B. Winter classification, impaction condition, and maximal mouth opening were assessed by a doctor. The Winter classification had two indexes representing the difficulty of tooth extraction: class classification (Pell and Gregory classification: I, II, and III) and position classification (A, B, and C) [13]. Impaction condition was assessed as either incomplete impaction, in which the tooth has penetrated the mucosa but is partially covered by bone or soft tissue, or as complete impaction, in which the tooth has not penetrated the mucosa. Maximal mouth opening was measured between the incisal edge of the upper and lower right central incisors when the patient opened his or her mouth upon request before tooth extraction.

Prior to surgery, patients completed a questionnaire on smoking and inflammation history. The smoking history included whether the patient had ever smoked. The inflammation history included the presence or absence of gingival pain and swelling around the lower third molar to be extracted prior to the first visit. This questionnaire is routinely carried out for patients undergoing third molar extraction at Shizuoka General Hospital.

### Outcomes

A diagnosis of dry socket was determined by the doctor who had carried out the surgery when patients reported spontaneous post-extraction pain that did not subside 1 to 3 days postoperatively [2, 3].

Spontaneous post-extraction pain was measured in the following manner. After surgery, patients returned to hospital on the first postoperative day (POD1), at which time they were given a form on which to record their pain responses and were instructed by their doctor on how to use it. Degree of pain was assessed based on two measures: A visual analog scale (VAS) that ranged from 0 (no pain) to 10 (worst pain), and the number of loxoprofen sodium analgesic tablets (60 mg/tablet) taken by patients. Responses for both measures were entered by patients for the operative day, POD1, POD3, and the day that the sutures were removed (POD7).

### Statistical analysis

Continuous variables are presented as mean and standard deviation, and categorical variables are presented as frequency and percentage. The Wilcoxon rank-sum test and the chi-square test were used to compare the background of the gauze-insertion group and the non-insertion group and to compare variables related to the occurrence of dry socket.

Comparisons of outcomes between the two groups were conducted using a logistic regression analysis for the binary outcome of the occurrence of dry socket and a linear regression analysis for the continuous outcome of the VAS value of spontaneous pain perception. In the logistic regression analysis, odds ratio (OR), 95% confidence interval (95% CI) based on the Wald test, and corresponding P-value were calculated. In the linear regression analysis, least-square means, 95% CI based on the Wald test, and corresponding P-value were calculated. Multivariate models were adjusted by variable in relation to the differences between the two groups and to the occurrence versus non-occurrence of dry socket. A P-value of < .05 was considered statistically significant. All analyses were performed using SAS version 9.4 (SAS Institute, Cary, NC, USA).

## Results

### Characteristics of patients

Table 1 shows the characteristics of the patients in the gauze-insertion group (n = 215) and the non-insertion group (n = 46). The proportion of male to female patients was higher in the gauze-insertion group (61.6%) than in the non-insertion group (39.1%) (p = 0.008). The distribution of the classes in the G. B. Winter classification was also different in the two groups (p = 0.002).

**Table 1:** Baseline characteristics of the gauze-insertion and non-insertion groups.

Variables		Category	Non-insertion group	Gauze-insertion group	*p* value*
Number of cases			46	215	
Age (SD)			38.3 (10.5)	37.7 (13.5)	0.799
Sex (%)		Male	18 (39.1)	132 (61.1)	0.008
		Female	28 (60.9)	83(38.9)	
Smoking history (%)		No	37 (80.4)	173 (80.5)	1.000
		Yes	9 (19.6)	42 (19.5)	
Inflammation history (%)		No	28 (60.9)	115 (53.7)	0.417
		Yes	18 (39.1)	99 (46.3)	
		Missing	0	1	
G. B. Winter classification (%)	Position classification	A	33 (71.7)	163 (75.8)	0.424
		B	13 (28.3)	46 (21.4)	
		C	0 (0.0)	6 (2.8)	
	Class classification	I	23 (50.0)	162 (75.3)	0.002
		II	19 (41.3)	47 (21.9)	
		III	4 (8.7)	6 (2.8)	
Maximal mouth opening before extraction (SD)			47.4 (5.8)	46.3 (6.0)	0.284
Impaction condition (%)		Complete impaction	11 (23.9)	45 (21.0)	0.694
		Incomplete impaction	35 (76.1)	169 (79.0)	
		Missing	0	1	

Abbreviation: SD, standard deviation.

* The *p* values were calculated using the Wilcoxon rank-sum test and chi-square test.

### Incidence of dry socket

Table 2 shows the occurrence of dry socket in relation to the other variables. Occurrence was lower in the gauze-insertion group (0.9%, 2/215) than in the non-insertion group (19.6%, 9/46) (p<0.001). Maximal mouth opening before extraction was larger in the gauze-insertion group (p = 0.03).

**Table 2:** Baseline characteristics with/without occurrence of dry socket.

Variables		Category	With dry socket	Without dry socket	*p* value*
Number of cases (%)			11	251	
Group		Non-insertion	9 (81.8)	37 (14.7)	<0.0001
		Gauze insertion	2 (18.2)	214 (85.3)	
Age (SD)			41.9 (8.8)	37.6 (13.1)	0.097
Sex (%)		Male	8 (72.7)	142 (56.6)	0.362
		Female	3 (27.3)	109 (43.4)	
Smoking history (%)		No	10 (90.9)	200 (80.0)	0.697
		Yes	1 (9.1)	50 (20.0)	
Inflammation history (%)		No	7 (63.6)	136 (54.6)	0.759
		Yes	4 (36.4)	113 (45.4)	
		Missing	0	2	
G. B. Winter classification (%)	Position classification	A	9 (81.8)	187 (74.8)	1
		B	2 (18.2)	57 (22.8)	
		C	0 (0.0)	6 (2.4)	
		Missing	0	1	
	Class classification	I	8 (72.7)	177 (70.8)	0.461
		II	2 (18.2)	64 (25.6)	
		III	1 (9.1)	9 (3.6)	
		Missing	0	1	
Maximal mouth opening before tooth extraction (SD)			43.1 (3.0)	46.6 (6.0)	0.03
Impaction condition (%)		Complete impaction	1 (9.1)	55 (22.1)	0.466
		Incomplete impaction	10 (90.9)	194 (77.9)	
		Missing	0	2	

Abbreviation: SD, standard deviation.

* The *p* values were calculated using the Wilcoxon rank-sum test and chi-square test.

A multivariate logistic regression analysis adjusting for sex, the class classification of the G. B. Winter classification, and maximal mouth opening indicated a lower incidence of dry socket in the gauze-insertion group (odds ratio 0.019, 95% CI 0.003–0.113, p<0.001).

### Degree of spontaneous post-extraction pain

**Fig 2:**
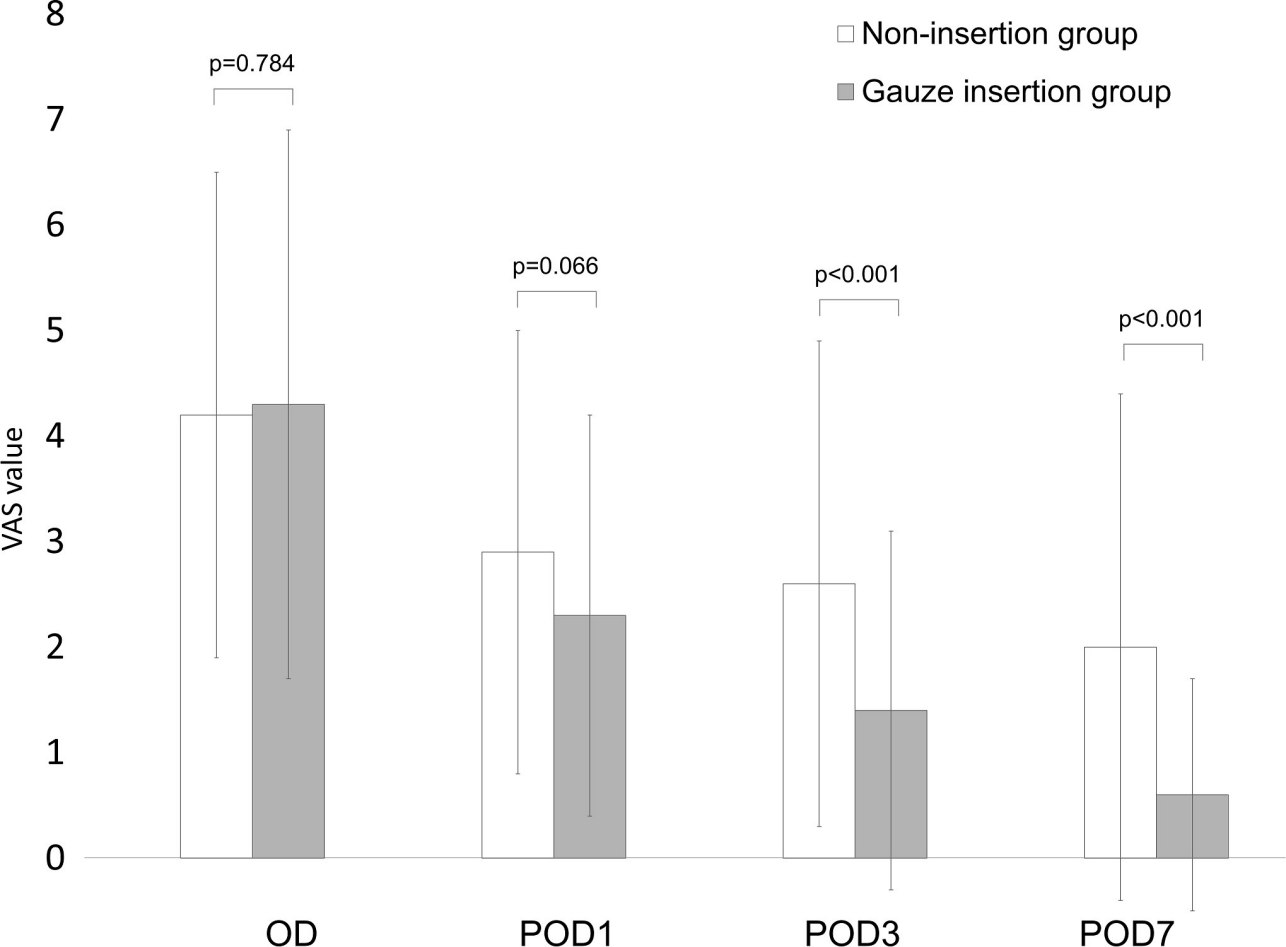
Changes in VAS value after tooth extraction in the gauze-insertion and non-insertion groups. Spontaneous post-extraction pain was assessed using a visual analogue scale (VAS) ranging from 0 (no pain) to 10 (worst pain) on four occasions: the operative day, postoperative day 1 (POD1), POD3, and at suture removal (POD7). Error bar indicates standard deviation. P-values were calculated using the Wilcoxon rank-sum test. Abbreviations: OD, operative day; POD1, postoperative day 1; POD3, postoperative day 3; POD7, postoperative day 7; VAS, visual analogue scale.

The degree of spontaneous post-extraction pain was evaluated with two measures: the VAS and the number of loxoprofen sodium oral analgesic tablets (60 mg/tablet) taken. Fig 2 shows changes in the VAS value after tooth extraction in the two groups. Statistical testing indicated that there was no difference in the VAS value between the two groups on the operative day (gauze-insertion group vs non-insertion group, mean [SD], 4.3 [2.6] vs 4.2 [2.3]) or on POD1 (2.9 [2.1] vs 2.3 [1.9]). On POD3, the VAS value of the gauze-insertion group (1.4 [1.7]) was lower than that of the non-insertion group (2.6 [2.3]) (p<0.001). Similarly, at suture removal on POD7, the VAS value of the gauze-insertion group (0.6 [1.1]) was lower than that of the non-insertion group (2.0 [2.4]) (p<0.001). Multivariate analysis showed that the VAS value of the gauze insertion group was lower on POD3 and POD7 (Table 3).

**Table 3:** VAS values for spontaneous post-extraction pain with regression model.

			Univariate regression model			Multivariate regression model*		
Variables		Category	Difference	95% CI	*p* value	Difference	95% CI	*p* value
VAS	POD1	Non-insertion group	0		0.077	0		0.211
		Gauze-insertion group	-0.57	-1.20–0.07		-0.42	-1.07–0.24	
	POD3	Non-insertion group	0.00		< .0001	0.00		0.001
		Gauze-insertion group	-1.17	-1.76 - -0.58		-1.08	-1.69- -0.47	
	POD7	Non-insertion group	0.00		< .0001	0.00		< .0001
		Gauze-insertion group	-1.42	-1.89 - -0.96		-1.39	-1.87 - -0.91	

Abbreviations: VAS, visual analogue scale; POD1, postoperative day 1; POD3, postoperative day 3; POD7, postoperative day 7; 95% CI, 95% confidence interval.

* The multivariate regression model was adjusted for sex, for the class classification of the G. B. Winter classification, and for maximal mouth opening.

**Fig 3:**
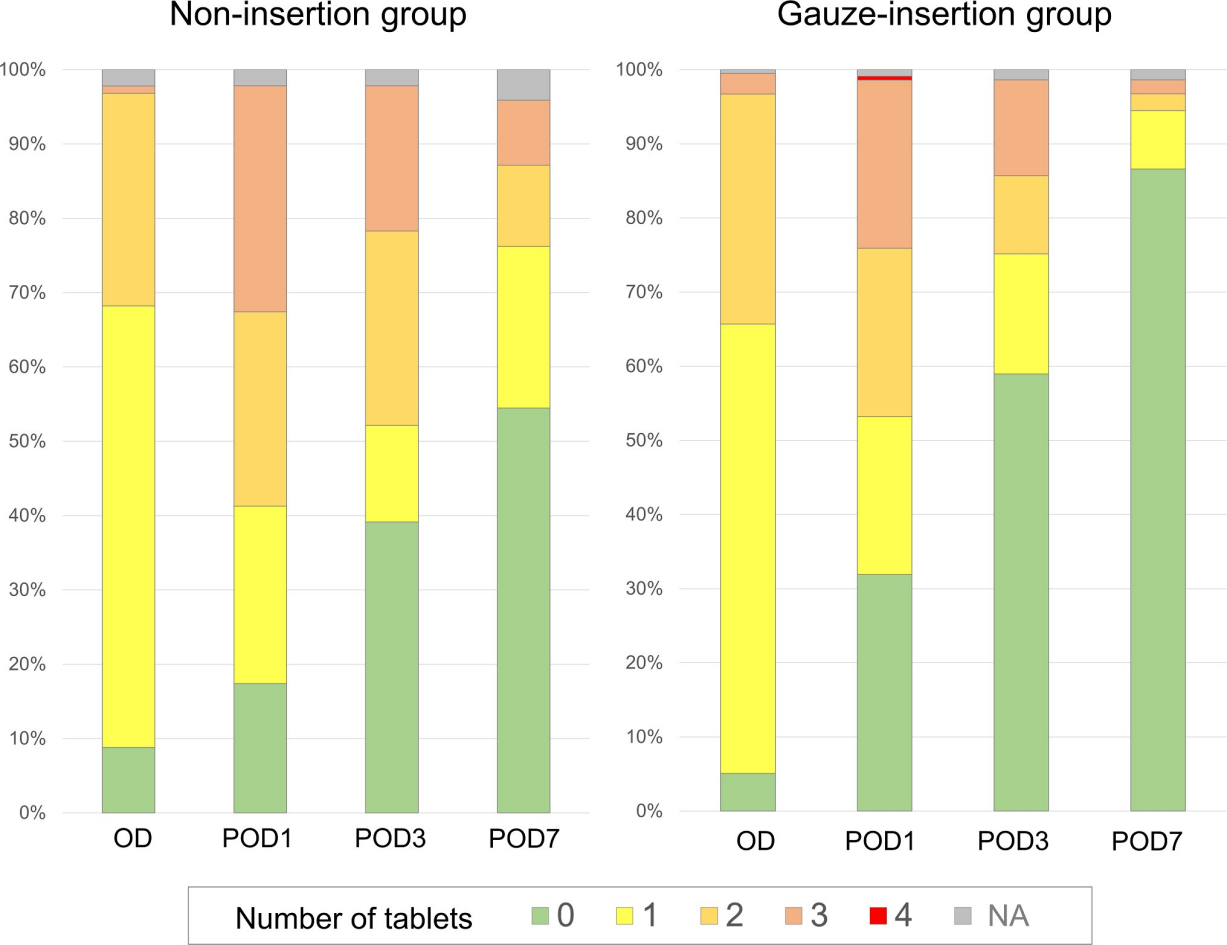
Number of oral analgesic tablets taken by each group. Patients reported the number of loxoprofen sodium oral analgesic tablets (60mg/tablet) they had taken on the operative day, on the first postoperative day (POD1), on POD3, and on the day of suture removal (POD7). Abbreviations: OD, operative day; POD1, etc., postoperative day 1, etc.

Fig 3 shows changes in the number of analgesic tablets taken by patients in the two groups after tooth extraction. Statistical testing indicated that there was no difference in the number of tablets taken on the operative day or on POD1. On POD3, the number of tablets taken by the two groups was different (p = 0.014), with the proportion of patients taking three tablets (the recorded maximum on POD3 and 7) being 13.1% (28/215) in the gauze-insertion group and 20% (9/46) in the non-insertion group. Similarly, with suture removal on POD7, there was a difference in the number of tablets taken by the two groups (p<0.001), with the proportion of patients taking three tablets being 1.9% (4/215) in the gauze-insertion group and 9.1% (4/46) in the non-insertion group.

## Discussion

The above results suggest that insertion of gauze coated with oxytetracycline-hydrocortisone ointment into the extraction socket immediately after third molar extraction reduces both the occurrence of dry socket and the degree of post-extraction pain. Dry socket was defined as the occurrence of spontaneous pain that did not subside 1 to 3 days after surgery, and the degree of spontaneous post-extraction pain was measured by VAS and by the number of oral analgesic tablets taken by patients. We found that the occurrence of dry socket was lower in the gauze-insertion group (0.9%) than in the non-insertion group (19.6%) (p<0.001). We also found that both the VAS value and the number of tablets taken was lower in the gauze-insertion group than in the non-insertion group on POD3 and POD7, indicating that spontaneous post-extraction pain was suppressed.

Dry socket results from the detachment of the blood clot from the extraction socket. This detachment can be caused both by the enhancement of the fibrinolytic system in the socket and by mechanical stimulation [6]. Hyperactivity of the fibrinolytic system is associated with local inflammation and with bacterial growth [14, 15]. Local inflammation is induced by invasive stimulation during surgery and by bacterial growth, both of which can lead to spontaneous post-extraction pain [16]. Mechanical irritation can be induced both by excessive rinsing of the mouth and by the invasion of food residue into the socket. Excessive rinsing not only stimulates the extraction wound, but can also lead directly to the dislodgement of the blood clot [4]. Invasion of food residue into the socket can also induce bacterial growth, followed by local inflammation [16, 17]. Mechanical irritation can also lead to post-extraction pain.

Oxytetracycline-hydrocortisone ointment consists of a combination of hydrocortisone and oxytetracycline hydrochloride. Hydrocortisone has a local anti-inflammatory effect, and oxytetracycline hydrochloride has an antibacterial effect and also suppresses fibrinolysis of the socket [18]. The addition of gauze helps protect the socket from mechanical stimulation and keeps the medicinal agents in the socket. Taken together, the above findings suggest that our procedure both prevents the occurrence of dry socket by keeping blood clots in the socket and suppresses spontaneous post-extraction pain.

Several studies on preventing the incidence of dry socket and post-extraction pain have been reported. A study on the preventive effect of chlorhexidine on dry socket reported that the incidence of dry socket was 7.5% for the topical administration of chlorhexidine gel into the socket and 25% for rinsing of the mouth with chlorhexidine mouthwash [7]. Another study using chlorhexidine gel reported that the VAS value decreased from 4.55 [SD 1.5] on POD1 to 1.50 [SD 1.46] on POD7 [19]. In several studies evaluating PRF, the incidence of dry socket was 9% [8] and the VAS value decreased from 4.27 [SD 2.75] on POD0 to 0.1 [SD 0.3] on POD7 [20]. Our procedure had a lower incidence of dry socket than any of the methods used in these previous studies, and also had the same or greater effect than those methods in reducing the degree of post-extraction pain. Furthermore, in our experience, the methods used in these previous studies have additional drawbacks. Chlorhexidine gel easily flows out of the socket and excessive rinsing of the mouth with chlorhexidine mouthwash can lead to the loss of blood clots, reducing the effectiveness of these methods, and the time required to prepare PRF makes it an inefficient treatment. There have also been reports of other methods of covering the socket and suppressing pain due to mechanical irritation with the use of periodontal bandage material or a protective splint [21]; however, these methods are costly and time-consuming.

A common treatment for dry socket is to insert ointment containing an antibacterial steroidal agent into the extraction socket and then cover the area with a surgical pack. One study showed that applying gauze coated with chlortetracycline ointment to the socket immediately after extraction was effective in preventing dry socket, but not in reducing pain [10]. In the present study, the procedure with hydrocortisone was effective not only in reducing the occurrence of dry socket, but also in reducing spontaneous post-extraction pain. In summary, this study suggests that our procedure utilizing hydrocortisone is more effective, simpler, and more economical than conventional procedures both for the prevention of dry socket and for the suppression of spontaneous post-extraction pain.

The present study has several limitations. First, it was an observational study, and the assignment of patients to the gauze-insertion and non-insertion groups was not randomized. We tried to improve the exchangeability between groups by adjusting for patient background factors. We did not investigate additional factors that might affect dry socket, such as bone mass, flap range, and operation time, but these factors are covered by the G. B. Winter classification [13, 22]. The influence of surgical skill on the occurrence of dry socket [23] is likely to be small, as all of the procedures were carried out by oral surgeons who had 5 or more years of experience and had been certified by the Japanese Society of Oral and Maxillofacial Surgeons. There have been several reports [24–26] that oral contraceptives increase the incidence of dry socket, but we could not adjust for this, as no patients in the non-insertion group were taking oral contraceptives. Second, it is unclear whether the decrease in the incidence of dry socket and spontaneous post-extraction pain are due to the effect of the combination of gauze and oxytetracycline-hydrocortisone ointment or to the effect of the ointment alone. We hypothesize that the use of gauze makes an independent contribution to these factors and suggest that further studies be carried out with randomized controlled trials.

## Conclusions

This study suggests that inserting gauze coated with oxytetracycline-hydrocortisone ointment into the extraction socket immediately after lower third molar extraction reduces the occurrence of both dry socket and spontaneous post-extraction pain.
